# Chlamydial virulence factor TarP mimics talin to disrupt the talin‐vinculin complex

**DOI:** 10.1002/1873-3468.13074

**Published:** 2018-05-15

**Authors:** Austin J. Whitewood, Abhimanyu K. Singh, David G. Brown, Benjamin T. Goult

**Affiliations:** ^1^ School of Biosciences University of Kent Canterbury UK

**Keywords:** adhesion, chlamydia, crystallography, molecular mimicry, talin, vinculin

## Abstract

Vinculin is a central component of mechanosensitive adhesive complexes that form between cells and the extracellular matrix. A myriad of infectious agents mimic vinculin binding sites (VBS), enabling them to hijack the adhesion machinery and facilitate cellular entry. Here, we report the structural and biochemical characterisation of VBS from the chlamydial virulence factor TarP. Whilst the affinities of isolated VBS peptides from TarP and talin for vinculin are similar, their behaviour in larger fragments is markedly different. In talin, VBS are cryptic and require mechanical activation to bind vinculin, whereas the TarP VBS are located in disordered regions, and so are constitutively active. We demonstrate that the TarP VBS can uncouple talin:vinculin complexes, which may lead to adhesion destabilisation.

## Abbreviations

**FP,** fluorescence polarisation

**HSQC,** heteronuclear single quantum coherence

**LD‐motif,** leucine‐aspartic acid motif

**TarP,** translocated actin recruitment protein

**VBS,** vinculin‐binding site

**Figure 1:**
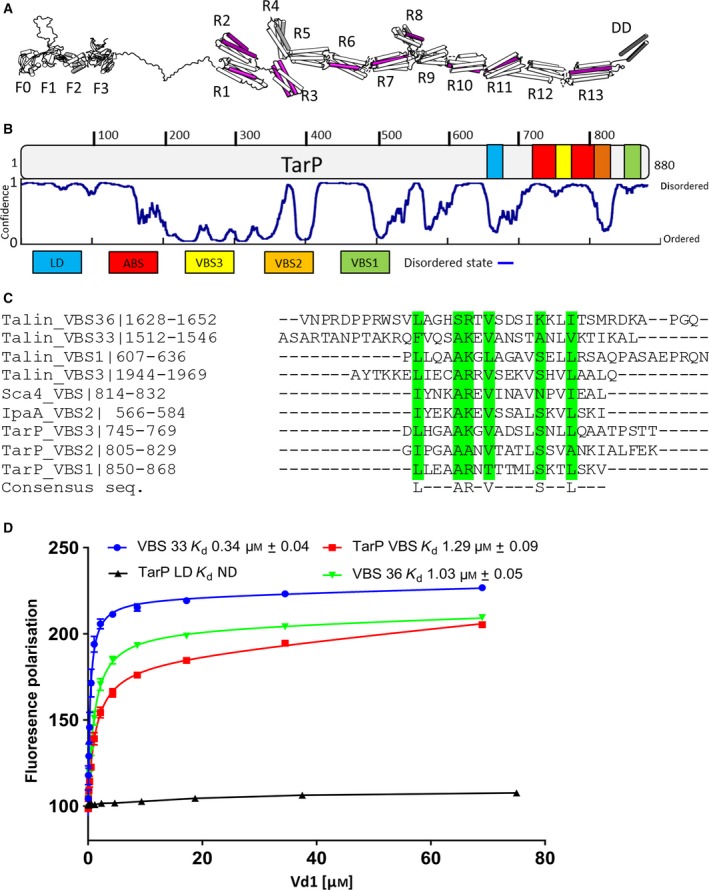
Biochemical characterisation of the TarP interaction with the vinculin head (Vd1). (A) Schematic of talin structure, with the location of the 11 talin VBS indicated (purple). (B) Schematic of TarP, indicating locations of VBS1 (green), VBS2 (orange), VBS3 (yellow), Actin binding site ABS (red) and LD‐motif (blue) at the C‐terminal. The disorder prediction trace generated using DISOPRED3 [44] is shown. (C) Multiple sequence alignment of vinculin binding sites, aligned using Clustal Omega. The consensus residues are highlighted in green. (D) Comparison of the Vd1:TarP and Vd1:talin interactions. Binding of fluorescein labelled talin VBS33, VBS36, TarP VBS (850–868)C and LD (655–680)C peptides to Vd1, measured using a fluorescence polarisation assay. Dissociation constants ± SE (μm) for the interactions are indicated in the legend. All measurements were performed in triplicate. ND, not determined.

Interactions between cells and the surrounding extracellular matrix (ECM), mediated *via* the integrin family of cell adhesion molecules, are fundamental to the development of multicellular life. These adhesions serve not just as attachment points but also as mechanosensitive signalling hubs, enabling cells to sense and respond to the external environment. Integrin receptors bound to ECM are coupled to the actin cytoskeleton *via* the mechanosensitive protein talin 1. Under force, helical bundles in the talin rod domain unfold, exposing multiple cryptic vinculin binding sites (VBS) 2, 3 that bind to the vinculin head (Vd1), activating autoinhibited vinculin by displacing the vinculin tail 4 (Fig. 1A). Activation of talin and vinculin also exposes numerous other cryptic binding sites for ligands that affect the assembly and regulation of both cell:ECM focal adhesions (FAs) and cell:cell junctions 5, 6.

These highly conserved attachment points linking the cell to the outside world have also become recognition sites for numerous infectious agents 7, 8 with some bacteria specifically targeting adhesion proteins for cellular entry. It has previously been shown that the *Shigella flexneri* effector protein IpaA 9 and the *Rickettsia* cell surface antigen Sca4 interact with vinculin 10. The atomic structures of these virulence factors reveal mimicry of the talin VBS. Thus, by forming amphipathic α‐helices that bind to Vd1, these virulence factors activate autoinhibited vinculin and hijack cell adhesion to aid pathogenesis.

Chlamydia invasion has been shown to require the effector protein translocated actin recruiting protein (TarP) which is thought to play an important role in actin recruitment 11, 12. TarP is translocated into the host cell by a chlamydial type 3 secretory system upon early elementary body (EB) attachment to the host cell. TarP injection by the bacteria leads to the recruitment and bundling of actin filaments at the point of invasion 12. Recently, Thwaites *et al*. reported the presence of a vinculin binding region in TarP, containing three vinculin binding sites (VBS) with ‘VBS1’ being essential for vinculin recruitment 13. Additionally, TarP was reported to contain a Leucine‐Aspartic acid motif (LD‐motif) with a similar consensus sequence to the second paxillin LD‐motif (LD2) which interacts with focal adhesion kinase (FAK) 14, and thus may provide TarP with a means of engaging FAK and altering cell adhesion signalling.

Here, we report the structure of the TarP:vinculin Vd1 complex and biochemically characterise and compare the interaction of the TarP VBS and talin VBS with vinculin. Whilst the affinities of the isolated VBS from TarP and talin are similar, their behaviour in larger polypeptide fragments is very different. Thus, the TarP VBS are positioned in unstructured regions, and TarP VBS1 is constitutively active whilst the talin VBS are buried inside folded rod domains and are cryptic. Furthermore, we demonstrate TarP VBS1 disrupts talin:vinculin complexes *in vitro*. This ability to uncouple vinculin from talin suggests that TarP and other virulence factors may have the capacity to trigger FA disassembly during invasion.

## Materials and methods

### Expression of recombinant polypeptides

Chicken vinculin Vd1 (residues 1–259), murine talin R10 (residues 1815–1973), and the FAK‐FAT domain (residues 941–1090) were cloned into a pET151 vector (Invitrogen) and expressed in *E .coli* BL21 (DE3) cells cultured in LB. Standard nickel‐affinity chromatography was used to purify the His‐tagged recombinant proteins as described previously 15. The proteins were further purified using anion‐exchange chromatography following cleavage of the 6xHis‐tag with TEV protease. Protein concentrations were determined using the respective extinction coefficients at 280 nm.

### Fluorescence polarisation assays

Peptides with either a C‐ or N‐terminal cysteine were synthesised by GLBiochem (China).

TarP_VBS1 (LLEAARNTTTMLSKTLSKV‐C; *C. caviae* residues 850–868)

TarP_LD (EGAEGLEHLLPQLRSHLDDAFDQQGN‐C; *C. caviae* residues 655–680)

Pax_LD2 (C‐NLSELDRLLLELN; paxillin residues 141–153)

Pax_LD4 (C‐ATRELDELMASLS; paxillin residues 262–274)

mTal1_VBS33 (C‐ASARTANPTAKRQFVQSAKEVANSTANLVKTIKAL; talin residues 1512–1546)

mTal1_VBS36 (C‐VNPRDPPRWSVLAGHSRTVSDSIKKLITSMRDKAP; talin residues 1622–1656)

Peptides were coupled to a thiol‐reactive fluorescein dye *via* the terminal cysteine. Stock solutions were made in phosphate‐buffered saline (PBS; 137 mm NaCl, 27 mm KCl, 100 mm Na_2_HPO_4_, 18  mm KH_2_PO_4_, pH 7.4), 1 mm TCEP and 0.05% Triton X‐100. Excess dye was removed using a PD‐10 desalting column (GE Healthcare, Chicago, IL, USA). Titrations were performed in PBS using a constant 1 μm concentration of fluorescein‐coupled peptide with increasing concentration of protein; final volume 100 μL in a black 96‐well plate. Fluorescent polarisation (FP) measurements were recorded on a BMGLabTech CLARIOstar plate reader at room temperature and analysed using GraphPad Prism. *K*_d_ values were calculated with nonlinear curve fitting using a one‐site total binding model.

### Analytical gel filtration

Gel filtration was performed using a Superdex‐75 size‐exclusion chromatography column (GE healthcare) at a flow rate of 1 mL·min^−1^ at 4 °C in 50 mm Tris pH 7.5, 150 mm NaCl, 2 mm DTT. A sample of 100 μL was run consisting of 100 μm of each protein/peptide, incubated at a 1 : 1 (talin:Vd1) or 1 : 1 : 1 (talin:Vd1:TarP VBS1/talin VBS36) ratio, at 37 °C for 30 min. In the competition experiment, an additional 30 nm of fluorescein‐coupled TarP peptide was added to visualise the TarP elution *via* absorbance at 494 nm. The elution absorbance was measured at three wavelengths: 220 nm, 280 nm and 494 nm (fluorescein absorbance). Elution was monitored by a Malvern Viscotek SEC‐MALS‐9 (Malvern Panalytical, Malvern, UK). Molar mass, refractive index and weight fraction (%) were determined using the OmniSEC software (Malvern Panalytical) and statistical significance assessed using a *T*‐test.

### X‐ray crystallography

Crystallisation trials for Vd1 in the presence of TarP VBS peptide were conducted at 21 °C by hanging drop vapour diffusion while maintaining a 1 : 1 protein to peptide ratio. Crystals were obtained in a condition containing 0.1 m sodium citrate tribasic dehydrate pH 5.6 and 20% v/v 2‐propanol. Crystals were cryoprotected in the same solution supplemented with 20% v/v glycerol prior to vitrification in liquid nitrogen. Diffraction dataset was collected at 100 K on beamline I03 at Diamond Light Source (Didcot, UK) using a Pilatus3 6M detector (Dectris, Baden, Switzerland). Crystallographic data were processed by autoPROC 16, which incorporates XDS 17, AIMLESS 18 and TRUNCATE 19 for data integration, scaling and merging. Structure of the Vd1/TarP complex was determined using chicken vinculin head as template (PDB: 3ZDL 20) for molecular replacement search carried out with PHASER 21. Manual model adjustment and refinement were performed with COOT 22 and REFMAC 23 respectively. Model was validated with MOLPROBITY 24 and interaction properties were determined by PISA 25. Figure preparation was carried out with PYMOL (Schrödinger LLC, Cambridge MA, USA). For data collection, phasing and refinement statistics, Table 1. The structure has been deposited to RCSB Protein Data Bank with accession code 6FQ4.

**Table 1:** X‐ray data collection and refinement statistics for TarP‐Vd1 complex. Data collected from a single crystal

Data collection	
Synchrotron and Beamline	Diamond Light Source; I03
Space group	*P*2_1_2_1_2
Molecule/a.s.u	1
Cell dimensions	
*a*,* b*,* c* (Å)	51.80, 66.87, 95.83
α, β, γ (°)	90, 90, 90
Resolution (Å)	95.83–2.9 (2.96–2.9)^a^
*R*_merge_	0.156 (0.806)
*I*/σ*I*	8.1 (2.5)
*CC(1/2)*	0.994 (0.903)
Completeness (%)	99.8 (99.9)
Redundancy	6.1 (6.3)
Refinement	
Resolution (Å)	2.9
No. reflections	7455 (519)
*R*_work_/*R*_free_	0.28/0.34
No. atoms	
Protein	2082
Water	3
*B*‐factors (Å^2^)	
Protein/Peptide	94.24/95.73
Water	84.04
R.m.s. deviations	
Bond lengths (Å)	0.010
Bond angles (°)	1.430
Ramachandran plot	
Favoured/allowed/outlier (%)	93/6/1
Rotamer	
Favoured/poor (%)	59.2/21.01
Molprobity scores	
Protein geometry	3.42 (37th)^b^
Clash score all atoms	29 (81st)^b^
PDB accession no.	6FQ4

a Values in parentheses are for highest‐resolution shell.

b Values in parentheses indicate percentile scores as determined by Molprobity.

### NMR Spectroscopy

NMR spectra were obtained using a Bruker AVANCE III 600 MHz spectrometer equipped with CryoProbe. Experiments were performed at 298 K in 20 mm sodium phosphate pH 6.5, 50 mm NaCl, 2 mm DTT with 5% (v/v) ^2^H_2_O. Ligand binding was evaluated from ^1^H,^15^N‐HSQC chemical shift changes using 130 μm ^15^N‐labelled FAK‐FAT domain. Peptides were added at a 3 : 1 peptide:protein ratio.

## Results

### Chlamydial VBS interacts with vinculin

It has previously been shown that the interaction between TarP and vinculin is required for Chlamydia infection 13. TarP was shown to contain three VBS with only the C‐terminal VBS, VBS1, being critical for TarP function (Fig. 1B). Multiple sequence alignment with the VBS of talin (Fig. 1C) confirmed the region that contains the vinculin head domain (Vd1) consensus binding motif LxxAAxxVAxxVxxLIxxA 26 as reported previously 13.

To evaluate how the interaction of Vd1 with the TarP VBS1 compares to its interaction with talin VBS, we measured the relative binding affinities using an *in vitro* fluorescence polarisation (FP) assay. In this assay, synthetic VBS peptides (Materials and methods) were fluorescently labelled with fluorescein and titrated against an increasing concentration of Vd1. Binding of the VBS peptide to Vd1 results in an increase in fluorescence polarisation (Fig. 1D), which can be used to determine the binding constant, *K*_d_. The TarP VBS1 peptide bound to Vd1 with an affinity of 1.29 μm. The talin VBS located on talin helices, 33 and 36 (VBS33 and VBS36), bound with comparable affinities of 0.34 μm and 1.03 μm respectively (Fig. 1D). The TarP LD region (residues 655–680), which does not interact with Vd1, was used as a negative control.

Although the affinity of the TarP VBS1 for vinculin is comparable to the VBS in talin, the location of the VBS are markedly different between the two proteins. Talin VBS are maintained in a cryptic conformation, buried inside the hydrophobic core of the talin rod domain bundles 27, and require exposure by mechanical force across talin to unfold the bundles 3, 28. In contrast, the VBS in TarP are situated in disordered regions of the molecule and are therefore likely to be constitutively active (Fig. 1B). The affinity of talin VBS in folded rod domains for Vd1 is significantly lower due to the energy required to unfold the domain to expose the VBS 29. However, this reduced affinity of talin for vinculin is rapidly overcome by force exerted on talin, an effect that is readily reversible when force is removed, meaning that the talin:vinculin interactions are exquisitely force‐dependent 28.Therefore, in the absence of mechanical force, TarP has the potential to outcompete folded talin to bind vinculin.

### The structure of TarP VBS1 in complex with the vinculin head

To further characterise the interaction between TarP VBS1 and vinculin, we crystallised a complex of TarP (850–868) with vinculin Vd1. The crystals containing one molecule of the complex in the asymmetric unit were in orthorhombic space group *P*2_1_2_1_2 and diffracted to a useful resolution of 2.9 Å.

**Figure 2:**
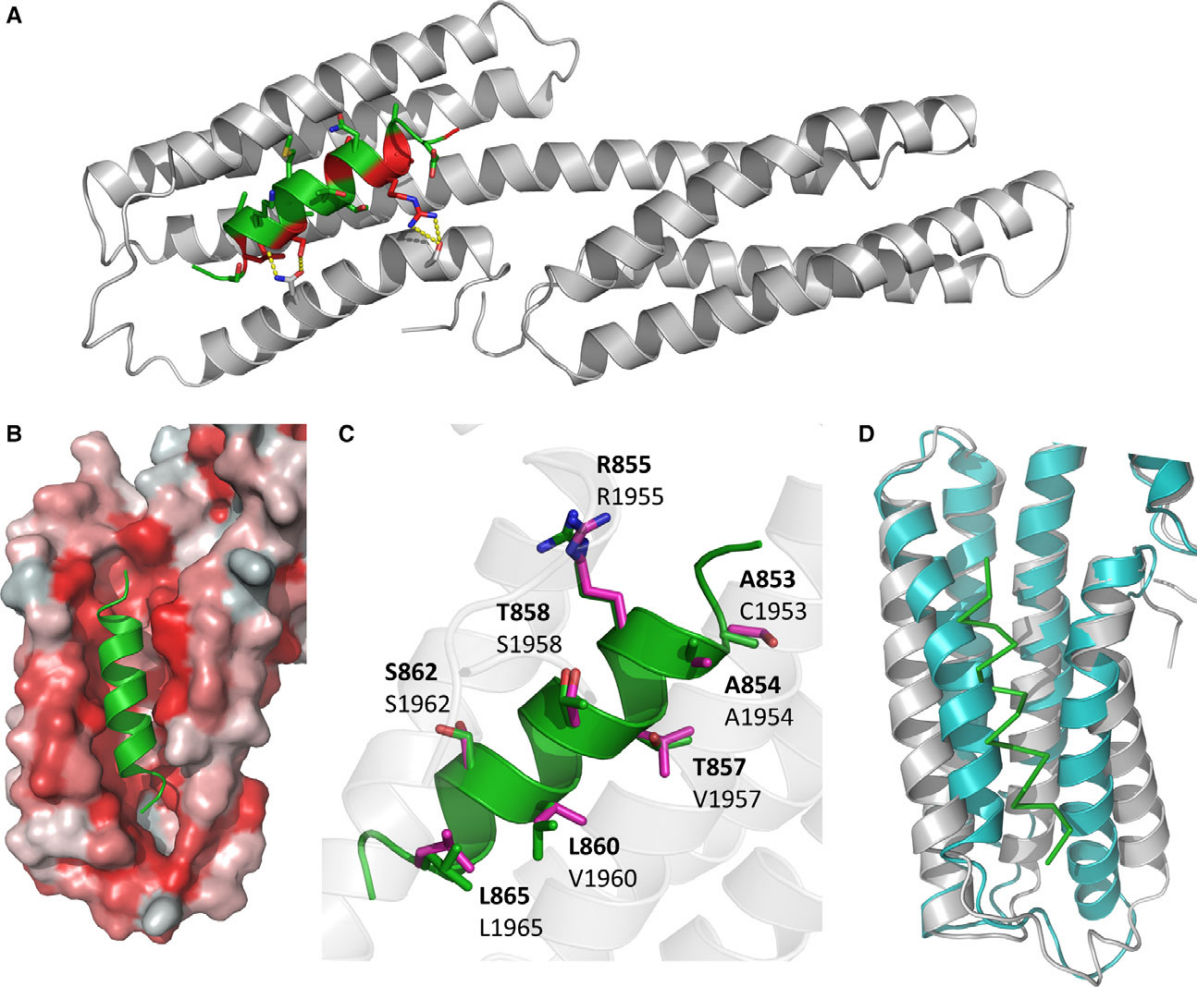
Crystal structure of TarP in complex with Vd1. (A) Cartoon representation of the complex of Vd1 (grey) bound to TarP VBS (green); the consensus VBS residues are shown in red. (B) TarP VBS (green) docks into a hydrophobic groove on Vd1. Vd1 is represented as surface coloured by hydrophobicity: hydrophobic = red, hydrophilic = white. (C) TarP VBS peptide (green) aligned with talin VBS46 peptide (purple, PDB:1RKC 4) with Vd1‐interacting sidechains from both VBS shown as sticks and TarP residues (top bold) and corresponding vbs46 residues are shown. (D) VBS binding causes conformational change in the Vd1 domain. Comparison of apo Vd1 (cyan, PDB:1TR2 [45]) and TarP bound Vd1 (grey). The TarP peptide is shown as a ribbon (green).

The structure of the complex was determined by molecular replacement (Fig. 2A; statistics in Table 1) and shows good agreement with the complexes of Vd1 with other VBS from talin 4, 30, sca‐4 31 and IpaA 32. The TarP VBS1 forms an α‐helix that embeds into the hydrophobic groove formed between α‐helices 1 and 2 of the Vd1 N‐terminal 4‐helix bundle, forming a structure that resembles a five‐helix bundle (Fig. 2B). Analysis of the complex interface by PISA indicated that 54.1% of the VBS surface area, including the consensus residues, is buried in the complex interface (Fig. 2A). Furthermore, two hydrogen bonds were identified: TarP Arg‐855 to Vd1 Ser‐11, and TarP Ser‐862 to Vd1 Gln‐18. Structural alignment of known VBS structures indicates that these hydrogen bonds are well conserved. Upon complex formation, TarP significantly alters the positions of Vd1 helices 1 and 2, widening the groove between the two and exposing the hydrophobic core (Fig. 2C), mimicking the way talin activates vinculin, causing the release of the vinculin tail 4, 33. With sidechains almost identical in length and character to talin VBS, TarP VBS1 is able to pack tightly into the Vd1 hydrophobic groove accounting for the high affinity we measured (Fig. 1C). The strong resemblance of the TarP VBS1 to the VBS in talin demonstrates the molecular mimicry employed by TarP to hijack the host cell adhesion machinery.

### The TarP peptide competes with talin for binding to vinculin

**Figure 3:**
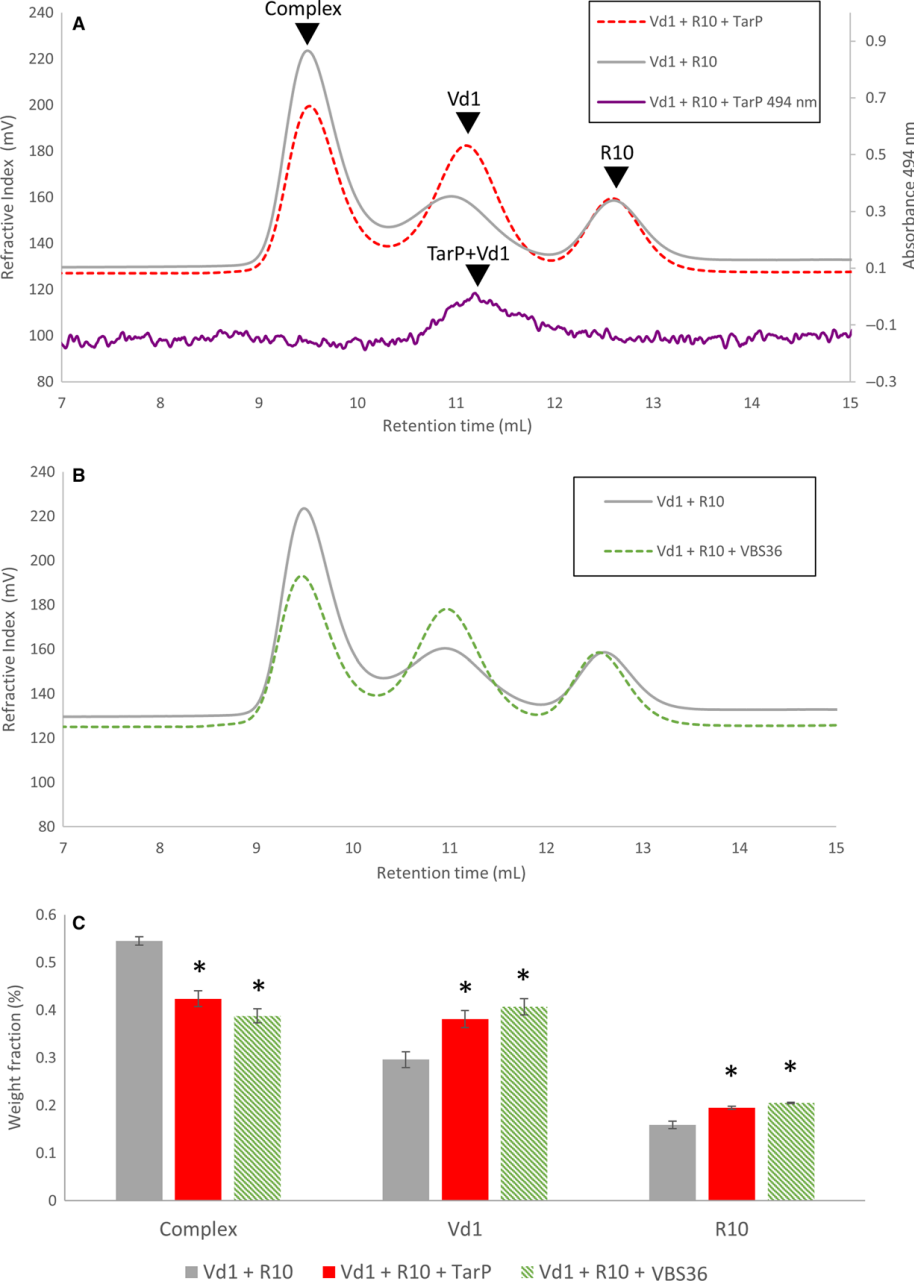
TarP VBS disrupts the interaction between talin R10 and vinculin Vd1. Vd1 was incubated with talin R10 at 37 °C for 30 min then analysed on a gel filtration column (grey). The experiment was repeated with the addition of a stoichiometric amount of TarP VBS peptide (A) and then with talin VBS36 (B). All experiments were done in triplicate. 1% fluorescein‐labelled TarP VBS peptide was added to monitor TarP VBS elution at 494 nm which confirmed that TarP eluted bound to Vd1 (purple). (C) the relative ‘Weight Fraction’ percentage for talin:vinculin complex, talin, vinculin peaks in the absence and presence of both TarP VBS and VBS36 peptides. Data are means ± SEM; *P < 0.05 by *T*‐test. Both peptides reduced the R10‐Vd1 complex.

Since TarP VBS1 binds to the same site on vinculin as the talin VBS, this raises the possibility that TarP binding might compete with talin for vinculin binding. A similar phenomenon was seen in *Drosophila* recently, where expression of a GFP‐VBS construct was found to disrupt talin:vinculin interactions *in vivo* 34. Using analytical gel filtration, we measured the interaction between Vd1 and a VBS‐containing talin helical bundle. We selected talin rod domain R10, which contains a single VBS (VBS46) 35. Equimolar amounts of Vd1 and talin R10 incubated together at 37 °C formed a 1 : 1 complex (Fig. 3A‐B). Adding a stoichiometric amount of TarP VBS1 peptide (Fig. 3A), or a peptide of an isolated talin VBS (VBS36; Fig. 3B) resulted in a significant reduction in the talin:Vd1 peak and concomitant increases in the monomer peaks of the respective proteins. To confirm that disruption of the talin:Vd1 complex was due to competition by the TarP VBS1 peptide, we spiked the TarP VBS1 peptide with 30 nm of fluorescein‐TarP VBS1 peptide (as used in the FP assay). The fluorescein‐coupled TarP VBS1 eluted in the same fractions as Vd1, confirming that the TarP peptide was bound to Vd1. To quantitate this competition, we used the SEC‐MALS OmniSEC software to determine the weight fraction (%) of each peak in Fig. 3A‐B and this analysis is shown in Fig. 3C. This demonstrates that TarP, and exposed VBS in general, can out‐compete talin for binding to Vd1 *in vitro* even when the vinculin:talin complex is already formed. Whilst the isolated talin VBS and TarP VBS1 peptides have similar affinities, the affinity of Vd1 for the intact talin rod domain is reduced significantly due to the cryptic nature of talin VBS in the folded talin helical bundles 29. As a consequence, the constitutively active nature of the TarP VBS allows it to disrupt vinculin:talin complexes. Vinculin binding to talin inhibits talin refolding 28 and is important for FA stabilisation. TarP disruption of this complex could lead to the loosening of adhesion by disrupting the talin:vinculin:actin cytoskeletal connection. This may mean that, as well as providing a means of entry and a mechanism to hijack the actin machinery, infection might also destabilise FAs at the point of entry.

### The TarP leucine‐aspartic acid motif

**Figure 4:**
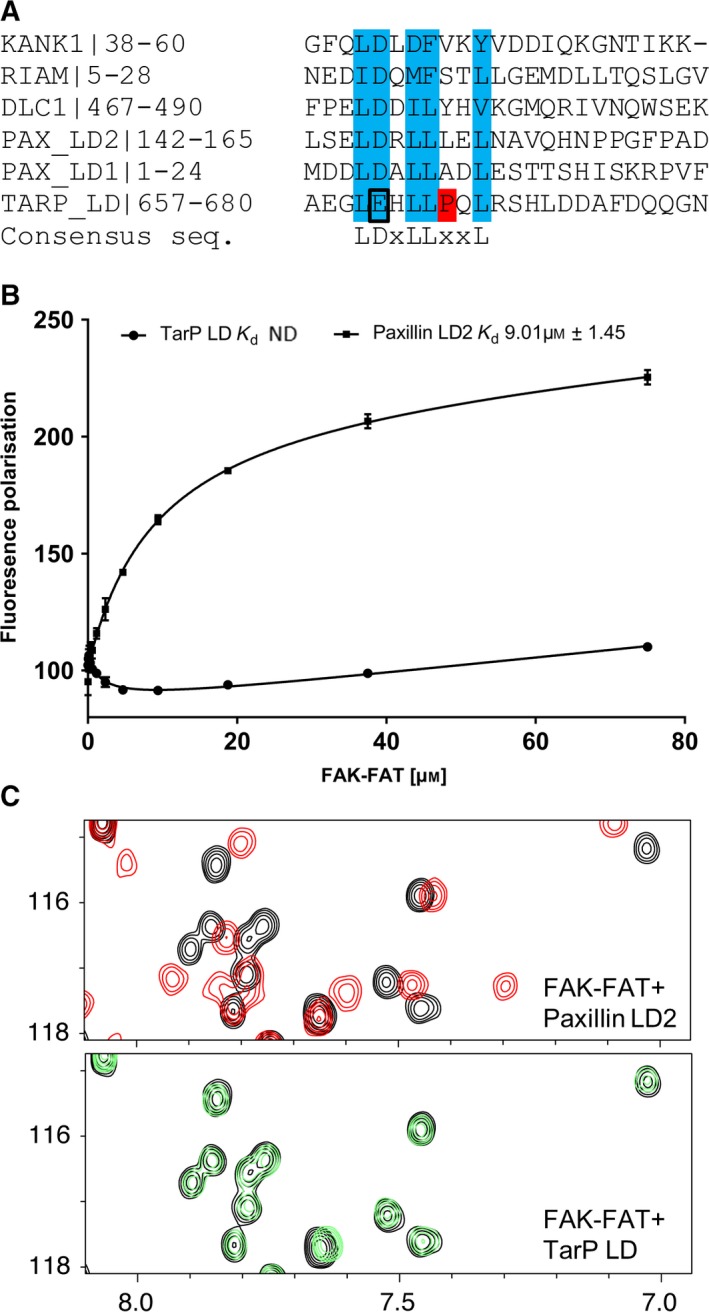
The TarP LD‐motif does not bind to FAK. (A) Multiple sequence alignment of known LD‐motifs and TarP, generated using Clustal Omega; the consensus binding residues are highlighted in blue. (B) Binding of fluorescein‐labelled TarP LD (655–680)C and Paxillin LD2 (141–153)C peptides to FAK‐FAT, measured using a fluorescence polarisation assay. Dissociation constants ± SE (μm) for the interactions are indicated in the legend. ND, not determined. (C) 1H,15N‐HSQC spectra of 130 μm 15N‐labelled FAK‐FAT in the absence (black) or presence of paxillin LD2 peptide (red; top panel) or TarP LD (green; bottom panel) at a ratio of 1 : 3.

Leucine‐Aspartic acid motifs (LD‐motifs) are well‐recognised protein:protein interaction motifs 36, first identified in the FA protein paxillin, and shown to be required for paxillin to interact with focal adhesion kinase (FAK) 37. The FAK–paxillin interaction was subsequently mapped to the focal adhesion targeting (FAT) domain of FAK 38. It was reported previously that TarP contains an LD‐motif (residues 655–680; TarP LD‐motif) with sequence homology to paxillin LD2 14, and that this LD‐motif interacts with the FAK‐FAT domain and plays a role in actin recruitment. The alignment of the TarP LD‐motif with the LD domains in KANK1 39, RIAM 40, DLC1 20 and the paxillin LD1 and LD2 motifs are shown in Fig. 4A. To investigate the interaction of TarP LD‐motif with FAK, we used the FP assay utilising fluorescein‐labelled LD‐motif peptides, and measured their binding to the FAK‐FAT domain. As expected, paxillin LD2 bound well to the FAK‐FAT domain (*K*_d_ ~ 9 μm) in line with previous reports 38. However, we observed no increase in polarisation with the TarP LD‐motif, suggesting that any interaction between TarP and FAK is too weak to be detected by the FP assay.

NMR is a powerful technique for studying interactions, even very weak (millimolar *K*_d_) interactions. Addition of paxillin LD2 peptide to ^15^N‐labelled FAK‐FAT resulted in large chemical shift changes indicative of a robust interaction (Fig. 4C). In contrast, addition of a threefold excess of TarP LD‐motif resulted in only very small shift changes, suggesting the peptide interacts only very weakly with FAK‐FAT (*K*_d_ > mm). This weak interaction explains the lack of binding in the FP experiment. The difference in binding affinity between the TarP LD‐motif and paxillin LD2 to FAK‐FAT may be explained by the presence of a proline residue, Pro675, in the middle of the TarP LD‐motif (Fig. 4A). It is likely the proline destabilises and/or causes a kink in the α‐helix formed by the TarP LD‐motif, but lack of binding might also be due to the substitution of glutamate for aspartate in the ‘LD’ region of the TarP LD‐motif. Therefore, despite the sequence homology, the TarP LD‐motif binds much weaker than paxillin‐LD2 to FAK‐FAT, further refining the specificity determinants of LD‐motifs and highlighting the fact that subtle changes in the sequence can significantly alter binding specificity.

It is possible that the TarP LD‐motif may bind to another, currently unrecognised LD‐binding domain protein, but it does not bind to FAK. Therefore, it seems likely that the TarP:FAK colocalisation reported previously *in cellulo* requires additional components that bring FAK and TarP together.

## Conclusions

In this study, we have further refined understanding of the molecular mechanisms underlying chlamydial infection *via* remodelling of the actin cytoskeleton; the ability of TarP to bind vinculin characterised here, and the recently characterised TarP WH2 motif that binds actin 41, look to be major components. Our data show that the constitutively active TarP VBS1 can out‐compete the mechanosensitive interaction between talin and vinculin. Vinculin is a key player in the regulation of FA dynamics 42 and cell:cell junctions 43, and the capacity of TarP VBS1 to uncouple vinculin‐mediated cytoskeletal connections during infection is therefore likely to have significant biological implications. Thus, it will be important to determine to what extent chlamydial infection alters the integrity and dynamics of cell:cell and cell:ECM junctions. Moreover, it raises the possibility that endogenous mammalian proteins might exist with constitutively active VBS, and that these could represent a new class of protein with the ability to regulate cell adhesion and migration.

## Author contributions

BTG conceived and supervised the study; AJW and BTG designed experiments; AJW and AKS performed experiments; BTG, AJW, AKS and DGB analysed data; BTG and AJW wrote the manuscript.
